# Complete Genome Sequence of Escherichia coli Siphophage BRET

**DOI:** 10.1128/MRA.01644-18

**Published:** 2019-01-31

**Authors:** Solange Ngazoa-Kakou, Yuyu Shao, Geneviève M. Rousseau, Audrey A. Addablah, Denise M. Tremblay, Geoffrey Hutinet, Nicolas Lemire, Pier-Luc Plante, Jacques Corbeil, Aristide Koudou, Benjamin K. Soro, David N. Coulibaly, Serge Aoussi, Mireille Dosso, Sylvain Moineau

**Affiliations:** a Plateforme de Biologie Moléculaire, Département Technique & Technologie, Institut Pasteur de Côte d’Ivoire, Abidjan, Côte d’Ivoire; b Département de biochimie, de microbiologie et de bio-informatique, Faculté des sciences et de génie, Université Laval, Québec, Canada; c College of Food Engineering and Nutritional Science, Shaanxi Normal University, Xi’an, China; d Félix d’Hérelle Reference Center for Bacterial Viruses and GREB, Faculté de médecine dentaire, Université Laval, Québec, Canada; e Department of Microbiology and Cell Science, University of Florida, Gainesville, Florida, USA; f Department of Molecular Medicine, Faculté de médecine, Université Laval, Québec City, Québec, Canada; University of Maryland School of Medicine

## Abstract

The lytic Escherichia coli siphophage BRET was isolated from a chicken obtained at a local market in Abidjan, Côte d’Ivoire. Its linear genome sequence consists of 59,550 bp (43.4% GC content) and contains 88 predicted genes, including 4 involved in archaeosine biosynthesis. Phage BRET is related (95% nucleotide identity) to Enterobacteria phage JenK1.

## Abstract (precis)

The lytic Escherichia coli siphophage BRET was isolated from a chicken obtained at a local market in Abidjan, Côte d’Ivoire. Its linear genome sequence consists of 59,550 bp (43.4% GC content) and contains 88 predicted genes, including 4 involved in archaeosine biosynthesis.

## ANNOUNCEMENT

**FIG 1:**
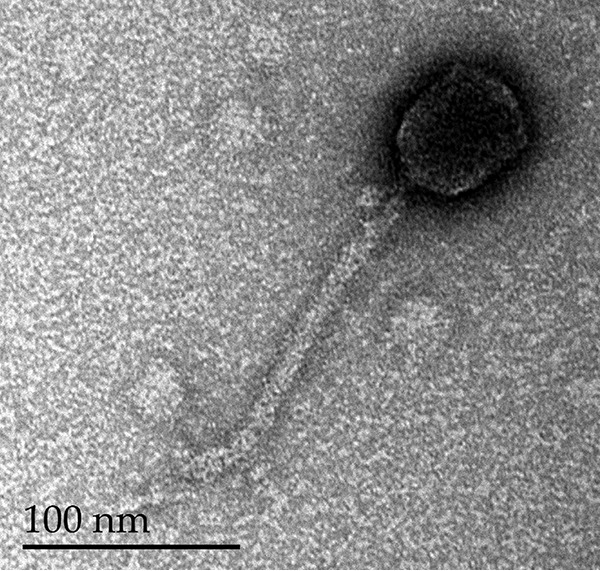
Micrograph of phage BRET stained with uranyl acetate (2%). Bar, 100 nm.

Phages are being reconsidered as complements to antibiotics in many countries (1–3). Here, a new virulent phage was isolated from the gastrointestinal tract of a chicken collected at a local market in Abidjan, Côte d’Ivoire. A 2-cm intestine sample was suspended in 2 ml saline and filtered (0.45 µm). The filtrate was added to Escherichia coli HER1036 in LB medium and incubated overnight at 37°C with shaking (4). A phage plaque was purified three times from LB plates and designated BRET. The lysate was observed under a transmission electron microscope, and BRET, with a slightly elongated capsid of 67 ± 2 nm × 51 ± 1 nm and a noncontractile tail of 176 ± 8 nm × 10 ± 1 nm (Fig. 1), belongs to the Siphoviridae family (5).

Phage genomic DNA was isolated from the lysate (6), and a DNA library was prepared (Nextera XT DNA library preparation kit). Sequencing was performed with an Illumina MiSeq system (250-nucleotide [nt] paired-end reads). The 557,108 reads were assembled with Ray v3.0.0 (7) using a k-mer length of 31, which led to a single contig with a coverage of 1,384×. Redundant contig ends were removed to produce the final complete genome. Functional genes were predicted with GeneMarkS (http://topaz.gatech.edu/genemark/genemarks.cgi), PECAAN Auto Annotation Tool (https://discover.kbrinsgd.org/autoannotate/), Geneious v11.0.5, and Glimmer v1.5 (plugin of Geneious v11.0.5) using the following principles: genes started with ATG, GTG, or TTG codons and were preceded with a Shine-Dalgarno (SD) sequence similar to AGGAGGU (5′ to 3′). Only coding sequences (CDS) with more than 30 amino acids were annotated with Blast2GO v5.2.1 (8) and the deduced proteins were searched for function using the NCBI nonredundant protein database and a cutoff *E* value of 0.001. tRNAs were searched using tRNAscan-SE v2.0 (http://lowelab.ucsc.edu/tRNAscan-SE/).

The phage BRET genome consists of 59,550 bp with a GC content of 43.4%. The average nucleotide identity between phage BRET and Enterobacteria phage JenK1 was 95.15% according to MUMmer analysis in JSpeciesWS (http://jspecies.ribohost.com/jspeciesws/#analyse). BRET is also related to Enterobacteria phages 9g (94.06% identity), JenP1 (93.07%), and JenP2 (93.73%), as well as to Salmonella phage SE1 (93.75%).

A total of 88 CDS were predicted, with sizes ranging from 138 bp to 3,261 bp. A majority of CDS started with ATG (94.32%), while start codons of GTG (3.41%) and TTG (2.27%) were also identified. ORF1 was designated based upon the annotation of phage JenK1 (9). Only 26 CDS were assigned a function, including terminase (ORF1 and ORF2), capsid protein (ORF6), and tail-related proteins (ORF12, ORF14, ORF16, ORF20, ORF23 and ORF25). Over 70% of the deduced BRET proteins have unknown functions.

tRNAs were not found, but genes involved in archaeosine DNA modification were identified in the BRET genome (10–13). ORF29 is likely a DpdA (proposed DNA ribosyltransferase), while ORF30 is similar to FolE (GTP cyclohydrolase), ORF31 to QueD (6-carboxytetrahydropterin synthase), ORF33 to QueC (7-cyano-7-deazaguanine synthase), and ORF35 to QueE (5-carboxy-deazaguanine synthase). These enzymes probably lead to DNA modifications, which may protect the phage genome from host endonucleases (12).

Phage BRET was deposited in the Félix d’Hérelle Reference Center for Bacterial Viruses (www.phage.ulaval.ca) under the number HER589.

### Data availability.

The complete genome sequence of E. coli phage BRET is available in GenBank under the accession number MK165087, and the raw data are in the SRA database under accession number PRJNA508515.
